# Two-Time Scale Virtual Sensor Design for Vibration Observation of a Translational Flexible-Link Manipulator Based on Singular Perturbation and Differential Games

**DOI:** 10.3390/s16111804

**Published:** 2016-10-28

**Authors:** Jinyong Ju, Wei Li, Yuqiao Wang, Mengbao Fan, Xuefeng Yang

**Affiliations:** School of Mechatronic Engineering, China University of Mining and Technology, Xuzhou 221116, China; jjy1991@126.com (J.J.); liweicumt@163.com (W.L.); wuzhi3495@cumt.edu.cn (M.F.); hopeasy@126.com (X.Y.)

**Keywords:** translational flexible-link manipulator, vibration observation, rigid-flexible coupling, linear-quadratic differential games, singular perturbation

## Abstract

Effective feedback control requires all state variable information of the system. However, in the translational flexible-link manipulator (TFM) system, it is unrealistic to measure the vibration signals and their time derivative of any points of the TFM by infinite sensors. With the rigid-flexible coupling between the global motion of the rigid base and the elastic vibration of the flexible-link manipulator considered, a two-time scale virtual sensor, which includes the speed observer and the vibration observer, is designed to achieve the estimation for the vibration signals and their time derivative of the TFM, as well as the speed observer and the vibration observer are separately designed for the slow and fast subsystems, which are decomposed from the dynamic model of the TFM by the singular perturbation. Additionally, based on the linear-quadratic differential games, the observer gains of the two-time scale virtual sensor are optimized, which aims to minimize the estimation error while keeping the observer stable. Finally, the numerical calculation and experiment verify the efficiency of the designed two-time scale virtual sensor.

## 1. Introduction

Nowadays, in automatic machines, handling operations, bio-engineering and space equipment, robot technology has widespread application [1,2,3,4]. With the development of modern machinery and equipment in the direction of low energy consumption and light-weight, the flexible manipulator has gained growing attention [5,6,7]. However, owing to the feeble rigidity and heavy deflection, the flexible manipulator easily produces elastic deformation and vibration during operation, which has a terrible effect on the position precision, stability, and service life of the whole system. Thus, it is of vital significance to investigate the vibration characteristics and active vibration control strategy of the flexible manipulator [8].

Recently, owing to the excellent mechanical-electrical coupling characteristics, the piezoelectric smart materials of lead zirconium titanate (PZT) have been widely applied in the field of vibration damping for flexible manipulators [9,10,11,12]. Under appropriate voltage control, the PZT actuators, which are bonded on the surface of the flexible manipulator, can generate shear forces to suppress the elastic deformation. In [13], a PZT actuator was used for the vibration suppression of a single-link flexible manipulator under the examination of the PZT actuator placement. For the same experiment platform, a positive position feedback controller was applied to the PZT actuators for the vibration damping in [14]. In another study [15], based on the simulated annealing, the optimal placements of multiple distributed PZT actuators are determined and the vibration control was arranged through a linear-quadratic-regulator controller. The superiority of PZT actuators in structural vibration control has been verified by many scholars. In order to optimize the bonding locations of the PZT actuators and constitute effective feedback control, the vibration signals of any points of the flexible manipulator should be obtained firstly. In the current research, the sensors, such as piezoelectric sensors and acceleration sensors, are often applied to measure the vibration signals [16,17]. Unfortunately, for the flexible manipulator, the introduction of additional position and speed sensors is bound to affect the dynamic characteristics of the whole system [18]. Additionally, in the actual operation, for the restriction of the bonding site, it is difficult to obtain the accurate vibration signals for the specified locations of the PZT actuators on the flexible manipulator, which influences the control effectiveness seriously. Moreover, the performance of the sensors is susceptible to environmental factors, which reduce the reliability of the whole system.

Therefore, virtual sensor technology is becoming a hotspot in the field of high-speed driven systems. The state observers are these devices or models which apply the system input and output signals to estimate the states of the system. Through comparing the system output and the output of the state observer and setting the error correction device, the observation error can converge and the closed loop observer can be formed. According the observation theory, it is an effective method to estimate the elastic vibration of the flexible manipulator by an observer. Through the use of self-recurrent wavelet neural networks, an adaptive observer [19] was proposed to estimate the actuator and link velocity information of a flexible-joint electrically-driven robot and the position tracking of the link was achieved. In [20], for the purpose of avoiding the installation of many sensors on the small mechanical structure in the traditional control methods, the feasibility of avoiding velocity measurements to control dexterous robots was analyzed. Similarly, in the field of robot assembly, Park [21] designed a quasi-static modal filter to estimate the fast time-varying vibration for the synchronous control of the static shape deformation and vibration of flexible load. Without the vibration measurement of the operated objects, the trajectory tracking control of two planar robots, which are used to move a flexible beam, was realized in [22]. Mosayebi [23] designed a nonlinear high-gain observer to estimate the elastic degrees of freedom of a flexible-link manipulator. However, the above methods have not been considered for the rigid-flexible coupling effect for the flexible manipulator system. With one observer applied for the simultaneous estimation of both the global motion of the rigid base and the elastic vibration of the flexible manipulator, those methods have many shortages, such as complicated design, heavy computational burden, and poor real-time transmitting capability. Actually, the motion of the translational flexible-link manipulator (TFM) consists of the rigid base motion and the elastic vibration of the flexible manipulator, and those two motions happen in two time scales [24].

Based on the above issues, in this paper the dynamic model of the TFM system is decomposed into the slow subsystem and the fast subsystem by the singular perturbation method. The slow subsystem expresses the global motion of the rigid base while the fast subsystem expresses the elastic vibration of the flexible manipulator. Then, based on the observation theory of state observer, a speed observer and a vibration observer are designed for the slow subsystem and the fast subsystem, respectively. Moreover, through the linear-quadratic differential games, the observer gains are optimized to minimize the observation error.

This paper is structured as follows: Section 2 shows the modeling and decomposition of the TFM system; Section 3 indicates the design and analysis of the two-time scale virtual sensor which is the main content; the effectiveness of the designed two-time scale virtual sensor is verified in Section 4; and finally, Section 5 summarizes this work.

## 2. Dynamic Modelling and Decomposition of the TFM

**Figure 1:**
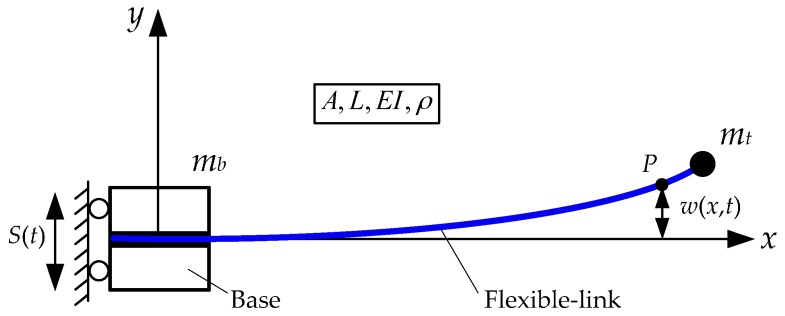
The translational flexible-link manipulator system.

The configuration of the TFM system is shown in Figure 1. During the modelling, the following assumptions are made: (1) the TFM system moves in the horizontal plane and the gravity and air resistance can be ignored; (2) since the ratio of the length and the cross-sectional area is large, the TFM is simplified as an Euler-Bernoulli beam and only the transverse vibration is taken into consideration.

The system parameters of the TFM are set as follows: *A*, *L*, *EI*, and *ρ* are the sectional area, the length, the bending stiffness, and the density of the flexible-link manipulator, respectively. *m_b_* and *S*(*t*) represent the mass and displacement of the base, respectively. *m_t_* denotes the mass of the payload. *ω*(*x*, *t*) indicates the transverse vibration of the flexible-link manipulator. The derivative notations are defined as: $\dot{\Delta }=\partial (\Delta )/\partial t$ and $\ddot{\Delta }=\partial^{2}(\Delta )/\partial t^{2}$.

Based on the assumption mode method [25,26], the transverse vibration of the TFM system can be expressed as:

(1) $$\begin{array}{cc} \omega (x,t) & =\sum_{i=1}^{m\to \infty }\phi_{i}(x)q_{i}(t)\\ & =\Phi (x)q(t) \end{array}$$

where *q*_i_(*t*) denotes the modal coordinates, *ϕ*_i_(*x*) denotes the *i*-th modal shape, and *m* is the modal number. Owing to the TFM can be considered as a cantilever beam, *ϕ*_i_(*x*) can be further represented as:

(2) $$\phi_{i}(x)=\text{sin}\;\beta_{i}x-\text{sinh}\;\beta_{i}x-\frac{\text{sin}\;\beta_{i}L+\text{sinh}\;\beta_{i}L}{\text{cosh}\;\beta_{i}L+\text{cos}\;\beta_{i}L}(\text{cos}\;\beta_{i}x-\text{cosh}\;\beta_{i}x)$$

where ${\beta_{i}}^{4}={\lambda_{i}}^{2}(\rho A/EI)$, and *λ_i_* is the *i*-th inherent frequency of the TFM.

The kinetic energy of the TFM system can be shown as:

(3) $$T=\frac{1}{2}m_{b}{\dot{S}}^{2}(t)+\frac{1}{2}\int_{0}^{L}\rho A{\left[\dot{S}(t)+\dot{\omega }(x,t)\right]}^{2}dx+\frac{1}{2}m_{t}{\left[\dot{S}(t)+\dot{\omega }(L,t)\right]}^{2}$$

The potential energy of the TFM system is caused by the elastic deformation of the flexible manipulator, which can be written as:

(4) $$U=\frac{1}{2}\int_{0}^{L}EI{\left[\frac{\partial^{2}\omega (x,t)}{\partial x^{2}}\right]}^{2}dx$$

The driving force of the base and the friction between the base and the guide constitute the external force of the TFM system. Then, the non-conservative work can be represented as:

(5) $$\delta W=F(t)\delta S(t)-\upsilon_{s}\dot{S}(t)\delta S(t)-\sigma_{q}\int_{0}^{L}\dot{\omega }(x,t)\sigma \omega (x,t)dx$$

where *F*(*t*) is the driving force of the base, *υ*_*s*_ is the friction coefficient between the base and the guide, and *σ_q_* is the structural damping of the TFM.

Based on the principle of the Euler-Bernoulli beam and Hamilton’s principle, the relationships among the kinetic energy, the potential energy, and the non-conservative work of the TFM system can be shown as:

(6) ∫(δT−δU+δW)dt=0

By substituting Equations (3)–(5) into Equation (6), the dynamics model of the TFM system can be expressed as:

(7) $$\left[\begin{array}{cc} M_{ss} & M_{sq}\\ M_{qs} & M_{qq} \end{array}\right]\left[\begin{array}{c} \ddot{S}\left(t\right)\\ \ddot{q}\left(t\right) \end{array}\right]+\left[\begin{array}{cc} p_{ss}\left(S,q,\dot{S},\dot{q}\right) & 0\\ 0 & p_{qq}\left(S,q,\dot{S},\dot{q}\right) \end{array}\right]\left[\begin{array}{c} \dot{S}\left(t\right)\\ \dot{q}\left(t\right) \end{array}\right]+\left[\begin{array}{cc} 0 & 0\\ 0 & K_{q} \end{array}\right]\left[\begin{array}{c} S\left(t\right)\\ q\left(t\right) \end{array}\right]=\left[\begin{array}{c} F\left(t\right)\\ 0 \end{array}\right]$$

where $M_{ss}\in {ℜ}^{1\times 1}$, $M_{sq}\in {ℜ}^{1\times m}$, $M_{qs}\in {ℜ}^{m\times 1}$, $M_{qq}\in {ℜ}^{m\times m}$, and $K_{q}\in {ℜ}^{m\times m}$. The variables and vector can be further represented as follows:

$$M_{ss}=m_{b}+\rho AL+m_{t}$$

$$p_{ss}\left(S,q,\dot{S},\dot{q}\right)=\upsilon_{s}$$

$$p_{qq}\left(S,q,\dot{S},\dot{q}\right)=diag\left[\begin{array}{cccc} \sigma_{q_{1}} & \sigma_{q_{2}} & \cdots & \sigma_{q_{m}} \end{array}\right]$$

$$M_{sq}=\rho A\int_{0}^{L}\Phi (x)dx+m_{t}\Phi (L)$$

$$M_{qs}=\rho A\int_{0}^{L}\Phi^{T}(x)dx$$

$$M_{qq}=diag\left[\begin{array}{cccc} \rho A & \rho A & \cdots & \rho A \end{array}\right]$$

$$K_{q}==diag\left[\begin{array}{cccc} \rho A{\lambda_{1}}^{2} & \rho A{\lambda_{2}}^{2} & \cdots & \rho A{\lambda_{m}}^{2} \end{array}\right]$$

According to the system characteristics, the quality matrix of the TFM is full-rank and the inverse matrix can be shown as:

(8) $$H\left(S,q\right)=\left[\begin{array}{cc} H_{ss}\left(S,q\right) & H_{sq}\left(S,q\right)\\ H_{qs}\left(S,q\right) & H_{qq}\left(S,q\right) \end{array}\right]={\left[\begin{array}{cc} M_{ss} & M_{sq}\\ M_{qs} & M_{qq} \end{array}\right]}^{-1}$$

Multiplying by **H**(*S*,**q**) on both sides, Equation (7) can be simplified as:

(9) $$\ddot{S}\left(t\right)=H_{ss}\left(S,q\right)\left[F\left(t\right)-p_{ss}\left(S,q,\dot{S},\dot{q}\right)\dot{S}\right]-H_{sq}\left(S,q\right)\left[p_{qq}\left(S,q,\dot{S},\dot{q}\right)\dot{q}+K_{q}q\right]$$

(10) $$\ddot{q}(t)=H_{qs}\left(S,q\right)\left[F\left(t\right)-p_{ss}\left(S,q,\dot{S},\dot{q}\right)\dot{S}\right]-H_{qq}\left(S,q\right)\left[p_{qq}\left(S,q,\dot{S},\dot{q}\right)\dot{q}+K_{q}q\right]$$

The next step is to define the singular perturbation parameter as $\varepsilon =\sqrt{1/K_{\text{min}}}$ ($K_{\text{min}}=\text{min}\left(K_{q}\right)$). Then, Equations (9) and (10) can be further converted as:

(11) $$\ddot{S}\left(t\right)=H_{ss}\left(S,\varepsilon^{2}\xi \right)\left[F\left(t\right)-p_{ss}\left(S,\varepsilon^{2}\xi ,\dot{S},\varepsilon^{2}\dot{\xi }\right)\dot{S}\right]-\varepsilon^{2}p_{qq}\left(S,\varepsilon^{2}\xi ,\dot{S},\varepsilon^{2}\dot{\xi }\right)\dot{\xi }-H_{sq}\left(S,\varepsilon^{2}\xi \right){\tilde{K}}_{q}\xi$$

(12) $$\varepsilon^{2}\ddot{\xi }=H_{qs}\left(S,\varepsilon^{2}\xi \right)\left[F\left(t\right)-p_{ss}\left(S,\varepsilon^{2}\xi ,\dot{S},\varepsilon^{2}\dot{\xi }\right)\dot{S}\right]-\varepsilon^{2}p_{qq}\left(S,\varepsilon^{2}\xi ,\dot{S},\varepsilon^{2}\dot{\xi }\right)\dot{\xi }-H_{qq}\left(S,\varepsilon^{2}\xi \right){\tilde{K}}_{q}\xi$$

where $\xi =q/\varepsilon^{2}$ and ${\tilde{K}}_{q}=\varepsilon^{2}K_{q}$.

When ε=0, the static solution of the vibration can be solved from Equation (12) and the variable expression is:

(13) $$\xi ={\left({\tilde{K}}_{q}\right)}^{-1}{\left[H_{qq}\left(S,0\right)\right]}^{-1}\left\{H_{qs}\left(S,0\right)\left[F\left(t\right)-p_{ss}\left(S,0,\dot{S},0\right)\dot{S}\right]\right\}$$

By substituting Equation (13) into Equation (11), the dynamic model of the slow subsystem can be obtained as:

(14) $$\begin{array}{cc} \ddot{S}\left(t\right) & =-\left(H_{ss}\left(S,0\right)-H_{sq}\left(S,0\right){\left[H_{qq}\left(S,0\right)\right]}^{-1}H_{qs}\left(S,0\right)\right)p_{ss}\left(S,0,\dot{S},0\right)\dot{S}\\ & +\left(H_{ss}\left(S,0\right)-H_{sq}\left(S,0\right){\left[H_{qq}\left(S,0\right)\right]}^{-1}H_{qs}\left(S,0\right)\right)F\left(t\right) \end{array}$$

According to the formulas for the block matrix inversion, one can obtain:

(15) $$H_{ss}\left(S,0\right)-H_{sq}\left(S,0\right){\left[H_{qq}\left(S,0\right)\right]}^{-1}H_{qs}\left(S,0\right)={\left[M_{ss}\right]}^{-1}$$

Based on the substitution of Equation (15) into Equation (14), the state space equation of the slow subsystem for the TFM system can be expressed as:

(16) $$\left[\begin{array}{c} {\dot{\alpha }}_{1}\\ {\dot{\alpha }}_{2} \end{array}\right]=\underset{A_{s}}{\underset{︸}{\left[\begin{array}{cc} 0 & 1\\ 0 & -M_{ss}^{-1}p_{ss}\left(S,0,\dot{S},0\right) \end{array}\right]}}\left[\begin{array}{c} \alpha_{1}\\ \alpha_{2} \end{array}\right]+\underset{B_{s}}{\underset{︸}{\left[\begin{array}{c} 0\\ M_{ss}^{-1} \end{array}\right]}}F(t)$$

where the state variables are defined as $\alpha =\left[\begin{array}{cc} \alpha_{1} & \alpha_{2} \end{array}\right]=\left[\begin{array}{cc} S & \dot{S} \end{array}\right]$.

For the fast subsystem of the TFM system, through defining the state variables as $z_{1}=\xi \left(t\right)$ and $z_{2}=\varepsilon \dot{\xi }\left(t\right)$, Equation (12) can be transformed into:

(17) $$\{\begin{array}{l} {\dot{z}}_{1}=z_{2}\\ \begin{array}{cc} \varepsilon {\dot{z}}_{2}= & H_{qs}\left(\alpha_{1},\varepsilon^{2}z_{1}\right)\left[F\left(t\right)-p_{ss}\left(\alpha_{1},\varepsilon^{2}z_{1},\alpha_{2},\varepsilon z_{2}\right)\alpha_{2}\right]-\\ & \varepsilon^{2}p_{qq}\left(\alpha_{1},\varepsilon^{2}z_{1},\alpha_{2},\varepsilon z_{2}\right)z_{2}-H_{qq}\left(\alpha_{1},\varepsilon^{2}z_{1}\right){\tilde{K}}_{q}z_{1} \end{array} \end{array}$$

In order to fix the model error between the slow subsystem and the actual system, the correction parameters of the boundary layer are introduced, which are defined as $\beta_{1}=\xi -\bar{\xi }=z_{1}-{\bar{z}}_{1}$ and $\beta_{2}=\varepsilon \dot{\xi }=z_{2}$. Defining the fast time scale as κ=t/ε, the slow variables can be regarded as constants on the edge of the boundary layer region, which means κ→0. Then, the state space equation of the fast subsystem for the TFM system is transformed from Equation (17) and can be expressed as:

(18) $$\left[\begin{array}{c} \frac{d\beta_{1}}{d\kappa }\\ \frac{d\beta_{2}}{d\kappa } \end{array}\right]=\underset{A_{f}}{\underset{︸}{\left[\begin{array}{cc} 0 & I\\ -H_{qq}\left(\alpha_{1},0\right){\tilde{K}}_{q} & 0 \end{array}\right]}}\left[\begin{array}{c} \beta_{1}\\ \beta_{2} \end{array}\right]+\underset{B_{f}}{\underset{︸}{\left[\begin{array}{c} 0\\ H_{qs}\left(\alpha_{1},0\right) \end{array}\right]}}F(t)$$

## 3. Two-Time Scale Virtual Sensor Design

**Figure 2:**
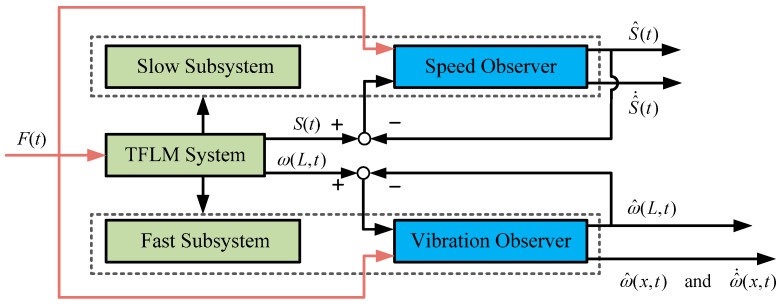
Structure diagram of the two-time scale virtual sensor.

With the analysis of the controllability and observability for the slow and fast subsystems of the TFM, the existence conditions of the observers are satisfied and the structure diagram of the two-time scale virtual sensor is illustrated in Figure 2, where $\hat{S}(t)$ and $\hat{\omega }(L,t)$ denote the estimates of the base displacement and the tip vibration of the flexible manipulator, respectively. $\hat{\omega }(x,t)$ and $\dot{\hat{\omega }}(x,t)$ express the estimates of ω(x,t) and $\dot{\omega }(x,t)$, where x∈[0,L].

Then, the speed observer equation of the slow subsystem and the vibration observer equation of the fast subsystem can be represented as:

(19) $$\dot{\hat{\alpha }}=A_{s}\hat{\alpha }+B_{s}F+K_{s}\left(S-\hat{S}\right)$$

(20) $$\dot{\hat{\beta }}=A_{f}\hat{\beta }+B_{f}F+K_{f}\left[\omega (L,t)-\hat{\omega }(L,t)\right]$$

where **K**_*s*_ and **K**_*f*_ indicate the observer gains of the speed observer and the vibration observer, respectively.

Subtracting Equations (19) and (20) from Equations (16) and (18), respectively, the error models of the two-time scale virtual sensor can be obtained as:

(21) $$\{\begin{array}{l} \dot{\tilde{\alpha }}(t)=A_{s}\tilde{\alpha }(t)-K_{s}\tilde{S}\\ \dot{\tilde{\beta }}(t)=A_{f}\tilde{\beta }(t)-K_{f}\tilde{\omega }(L,t) \end{array}$$

where $\tilde{\Delta }$ represents the observation error of the corresponding variables.

Based on the quadratic performance index, the linear-quadratic differential games can optimize the control parameters of the both sides of the differential games. Taking the state variables as $\tilde{x}={\left[\begin{array}{cc} \tilde{\alpha } & \tilde{\beta } \end{array}\right]}^{T}$, Equation (21) is transformed as the general form of the linear-quadratic differential games which is shown as Equation (22). **u**(*t*) and **v**(*t*) represent the control vectors of the speed observer equation and the vibration observer equation, respectively. Then, treating **u**(*t*) and **v**(*t*) as two sides of the countermeasures, the observer gains of the speed observer and the vibration observer are optimized by the linear-quadratic differential games.

(22) $$\{\begin{array}{l} \dot{\tilde{x}}(t)=A_{w}\tilde{x}(t)+C_{s}u(t)+C_{f}v(t)\\ \tilde{x}(0)=0 \end{array}$$

where $A_{w}=\left[\begin{array}{cc} A_{s} & 0\\ 0 & A_{f} \end{array}\right]$, $C_{s}=\left[\begin{array}{c} I_{2*2}\\ 0_{2m*2} \end{array}\right]$, $C_{f}=\left[\begin{array}{c} 0_{2*2m}\\ I_{2m*2m} \end{array}\right]$, $u(t)=-K_{s}\tilde{S}$, $v(t)=-K_{f}\tilde{\omega }(L,t)$.

The pay function is defined as:

(23) $$\begin{array}{cc} J(\tilde{x}(t),\tilde{\alpha }(t),\tilde{\beta }(t),u(t),v(t))= & {\left(\tilde{x}(T_{0})-\zeta \right)}^{T}W\left(\tilde{x}(T_{0})-\zeta \right)+\\ & \int_{0}^{T_{0}}\left[{\tilde{\alpha }}^{T}(t)Q_{1}\tilde{\alpha }(t)+{\tilde{\beta }}^{T}(t)Q_{2}\tilde{\beta }(t)+u^{T}(t)R_{1}u(t)+v^{T}(t)R_{2}v(t)\right]dt \end{array}$$

where $\zeta \in {ℜ}^{(2m+2)*1}$ expresses arbitrary given column vectors, $W\in {ℜ}^{\left(2m+2\right)(2m+2)}$ is a constant symmetric matrix, **Q**_1_ and **R**_1_ are symmetric positive definite matrices whose order is 2, and **Q**_2_ and **R**_2_ are symmetric positive definite matrices whose order is 2 m.

The Hamiltonian function of the error model of the two-time scale virtual sensor is defined as:

(24) $$\begin{array}{cc} H\left(\tilde{\alpha },\tilde{\beta },u,v,{\tilde{\alpha }}_{0},{\tilde{\beta }}_{0}\right)= & {\tilde{\alpha }}^{T}(t)Q_{1}\tilde{\alpha }(t)+{\tilde{\beta }}^{T}(t)Q_{2}\tilde{\beta }(t)+u^{T}(t)R_{1}u(t)+v^{T}(t)R_{2}v(t)+\\ & {\tilde{\alpha }}^{T}(t)\left(A_{s}^{T}P_{1}+P_{1}A_{s}\right)\tilde{\alpha }(t)+{\tilde{\beta }}^{T}(t)\left(A_{f}^{T}P_{2}+P_{2}A_{f}\right)\tilde{\beta }(t)+\\ & {\tilde{\alpha }}^{T}(t)P_{1}u(t)+u^{T}(t)P_{1}\tilde{\alpha }(t)+{\tilde{\beta }}^{T}(t)P_{2}v(t)+v(t)P_{2}\tilde{\beta }(t) \end{array}$$

Then, for the Hamiltonian function, the sufficient conditions for the existence of the extreme value are represented as:

(25) $$\{\begin{array}{l} \frac{\partial H}{\partial u}=2P_{1}\tilde{\alpha }(t)+2R_{1}u(t)=0\\ \frac{\partial H}{\partial v}=2P_{2}\tilde{\beta }(t)+2R_{2}v(t)=0 \end{array}$$

From Equation (25), the optimal observer gains of the two-time scale virtual sensor can be deduced as Equation (26) and the pay functional is minimized at this point:

(26) $$\{\begin{array}{l} u^{\gamma }(t)=-{R_{1}}^{-1}P_{1}\tilde{\alpha }(t)\\ v^{\gamma }(t)=-{R_{2}}^{-1}P_{2}\tilde{\beta }(t) \end{array}$$

Owing to $\frac{\partial^{2}H}{\partial u^{2}}=2R_{1}＞0$ and $\frac{\partial^{2}H}{\partial v^{2}}=2R_{2}＞0$, the minimum of the Hamiltonian function exists. Additionally, when **u**(*t*) and **v**(*t*) satisfy Equation (26), the minimum of the Hamiltonian function is 0.

Substituting Equation (26) into Equation (24) leads to:

(27) $$\begin{array}{cc} H\left(\tilde{\alpha },\tilde{\beta },u^{\gamma },v^{\gamma },{\tilde{\alpha }}_{0},{\tilde{\beta }}_{0}\right) & ={\tilde{\alpha }}^{T}(t)\left[A_{s}^{T}P_{1}+P_{1}A_{s}-P_{1}{R_{1}}^{-1}P_{1}+Q_{1}\right]\tilde{\alpha }(t)+\\ & {\tilde{\beta }}^{T}(t)\left[A_{f}^{T}P_{2}+P_{2}A_{f}-P_{2}{R_{2}}^{-1}P_{2}+Q_{2}\right]\tilde{\beta }(t)\\ & =0 \end{array}$$

Then, the satisfied Riccati functions of **P**_1_ and **P**_2_ can be obtained as:

(28) $$A_{s}^{T}P_{1}+P_{1}A_{s}-P_{1}{R_{1}}^{-1}P_{1}+Q_{1}=0$$

(29) $$A_{f}^{T}P_{2}+P_{2}A_{f}-P_{2}{R_{2}}^{-1}P_{2}+Q_{2}=0$$

In order to guarantee the stability of the two-time scale virtual sensor system under the action of the above optimal observer gains, the Lyapunov function of the error models is defined as:

(30) $$V\left(\tilde{\alpha }(t),\tilde{\beta }(t)\right)={\tilde{\alpha }}^{T}(t)P_{1}\tilde{\alpha }(t)+{\tilde{\beta }}^{T}(t)P_{2}\tilde{\beta }(t)$$

With Equations (28) and (29) combined, differentiating $V\left({\tilde{\alpha }}^{T}(t),\tilde{\beta }(t)\right)$ with respect to time yields:

(31) $$\begin{array}{cc} \dot{V}\left({\tilde{\alpha }}^{T}(t),\tilde{\beta }(t)\right) & ={\dot{\tilde{\alpha }}}^{T}(t)P_{1}\tilde{\alpha }(t)+{\tilde{\alpha }}^{T}(t)P_{1}\dot{\tilde{\alpha }}(t)+{\dot{\tilde{\beta }}}^{T}(t)P_{2}\tilde{\beta }(t)+{\tilde{\beta }}^{T}(t)P_{2}\dot{\tilde{\beta }}(t)\\ & ={\left[A_{s}\tilde{\alpha }(t)+u(t)\right]}^{T}P_{1}\tilde{\alpha }(t)+{\tilde{\alpha }}^{T}(t)P_{1}\left[A_{s}\tilde{\alpha }(t)+u(t)\right]+\\ & \;\;{\left[A_{f}\tilde{\beta }(t)+v(t)\right]}^{T}P_{2}\tilde{\beta }(t)+{\tilde{\beta }}^{T}(t)P_{2}\left[A_{f}\tilde{\beta }(t)+v(t)\right]\\ & ={\tilde{\alpha }}^{T}(t)\left[A_{s}^{T}P_{1}+P_{1}A_{s}-P_{1}{R_{1}}^{-1}P_{1}+Q_{1}\right]\tilde{\alpha }(t)+\\ & \;\;{\tilde{\beta }}^{T}(t)\left[A_{f}^{T}P_{2}+P_{2}A_{f}-P_{2}{R_{2}}^{-1}P_{2}+Q_{2}\right]\tilde{\beta }(t)-{\tilde{\alpha }}^{T}(t)Q_{1}\tilde{\alpha }(t)-\\ & \;\;{\tilde{\beta }}^{T}(t)Q_{2}\tilde{\beta }(t)-u^{T}(t)R_{1}u(t)-v^{T}(t)R_{2}v(t) \end{array}$$

Since **Q**_1_, **Q**_2_, **R**_1_, and **R**_2_ are symmetric positive definite matrices, one can obtain:

(32) $$\dot{V}\left({\tilde{\alpha }}^{T}(t),\tilde{\beta }(t)\right)=-{\tilde{\alpha }}^{T}(t)Q_{1}\tilde{\alpha }(t)-{\tilde{\beta }}^{T}(t)Q_{2}\tilde{\beta }(t)-u^{T}(t)R_{1}u(t)-v^{T}(t)R_{2}v(t)＜0$$

Thus, the designed two-time scale virtual sensor system is asymptotically stable.

## 4. Simulation and Experiment Validation

In this section, a simulation is employed to analyze the observation effect of the vibration observer for the elastic modes of the TFM. The material properties are defined as follows: *L* = 400 mm, *ρ* = 2.03 × 10^−^^3^ g/mm^3^, *A* = 135 mm^2^, and *E* = 25 GPa. As indicated in [27], only the first several low-order modes play a major role in the vibrations of the flexible manipulator. Thus, in order to simplify the analysis process, the first-order and second-order modes are given consideration. The total simulation time is 0.6 s and the simulation step is 0.001 s. The motion of the base is defined as a constant acceleration when t = 0–0.4 s and a constant speed when t = 0.4–0.6 s. The observer gains are set to $K_{s}={\left[\begin{array}{cc} 20.1 & 100.2 \end{array}\right]}^{T}$ and $K_{f}={\left[\begin{array}{cccc} 1.24E+3.0 & 9.30E+4.0 & 3.20E+5.0 & 3.3E+7.0 \end{array}\right]}^{T}$.

**Figure 3:**
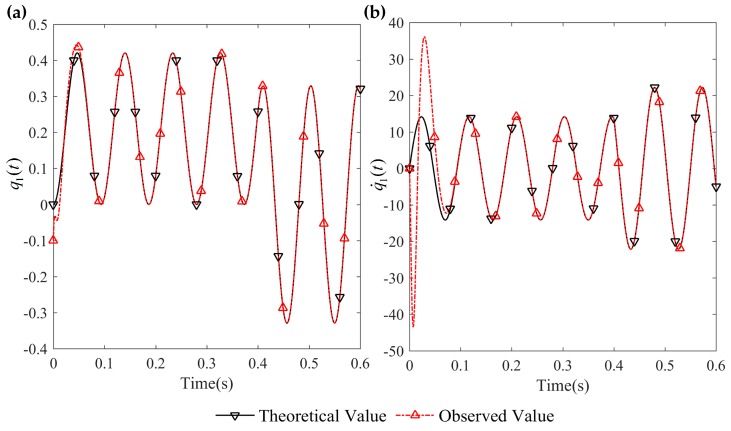
Tracking effect of the first-order mode and its derivative: (**a**) first-order mode tracking; (**b**) derivative of first-order mode tracking.

**Figure 4:**
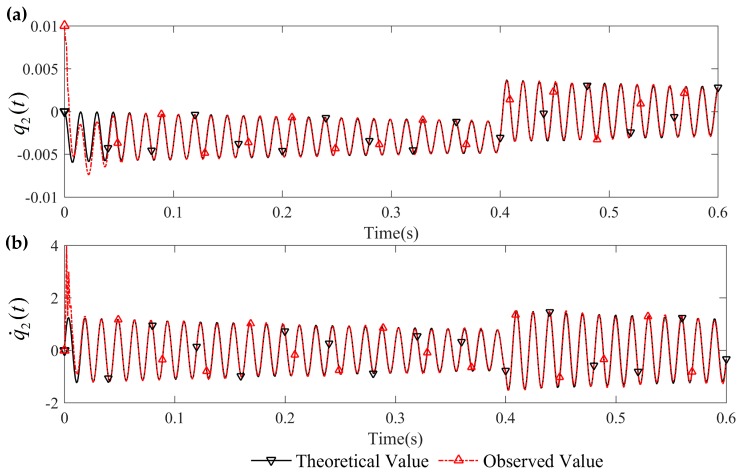
Tracking effect of the second-order mode and its derivative: (**a**) second-order mode tracking; (**b**) derivative of second-order mode tracking.

**Figure 5:**
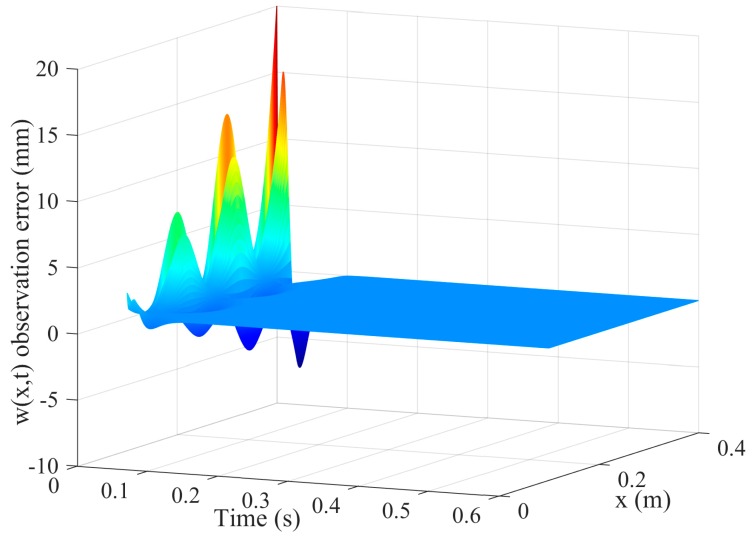
Tracking error of the vibration displacement of the TFM.

**Figure 6:**
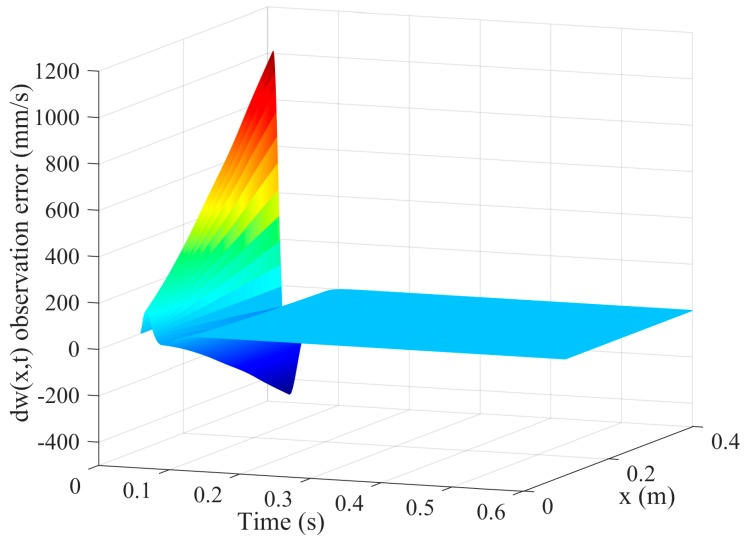
Tracking error of the vibration velocity of the TFM.

Figure 3 and Figure 4 are the tracking effect of the designed vibration observer for the first-order and second-order mode coordinates of the flexible-link manipulator. It is found that the initial error can be quickly reduced and the vibration modes and their derivative of the flexible-link manipulator are tracked with zero steady-state error. As can be seen from Figure 5 and Figure 6, the tracking error of the vibration displacement and velocity for any points of the flexible-link manipulator gradually tend to zero, under the base movement of constant acceleration and constant speed. The effectiveness of the proposed vibration observer is verified.

**Figure 7:**
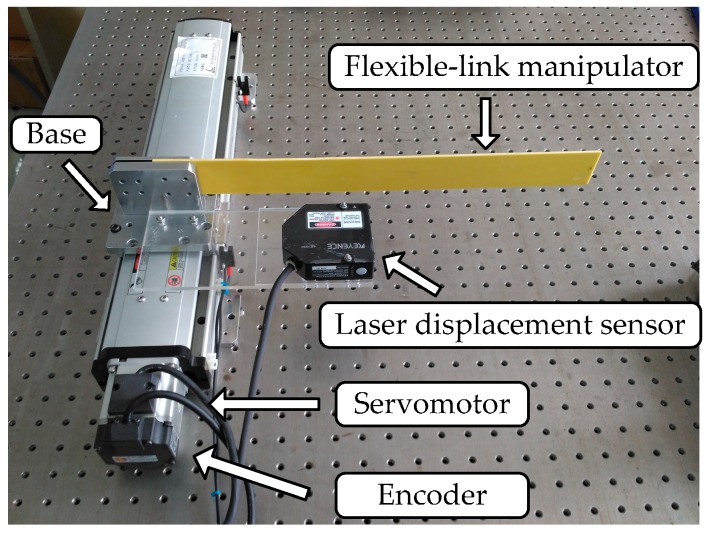
Photograph of the experimental setup.

Then, Figure 7 illustrates a photograph of the experimental device for the translational flexible-link manipulator. An epoxy resin plate of 400 mm length, 40 mm width, and 3 mm thickness is seen as a flexible-link manipulator whose vibration displacement is measured by a laser displacement sensor (KEYENCE: LK-G30, Osaka, Honshu, Japan) and the repetitive precision is 0.05 × 10^−3^ mm. The base of the flexible-link manipulator system is driven by a permanent magnet synchronous AC servomotor (Panasonic: NO. MSMJ022G1U, Osaka, Honshu, Japan) through a ball screw. The displacement of the base is transformed from the motor angle, which is measured by its rotary incremental encoder and the resolution of the encoder is 2^20^. The motion of the servomotor is controlled by a motion control card (GOOGOLTECH: NO: GT-400-SV-PCI, Hong Kong, China). The base motion is set as a point-to-point movement and the speed curve is set as a trapezoidal curve. The specific motion parameters of the base are as follows: the displacement is 200 mm, the maximum velocity is 100 mm/s, and the acceleration is 500 mm/s^2^.

**Figure 8:**
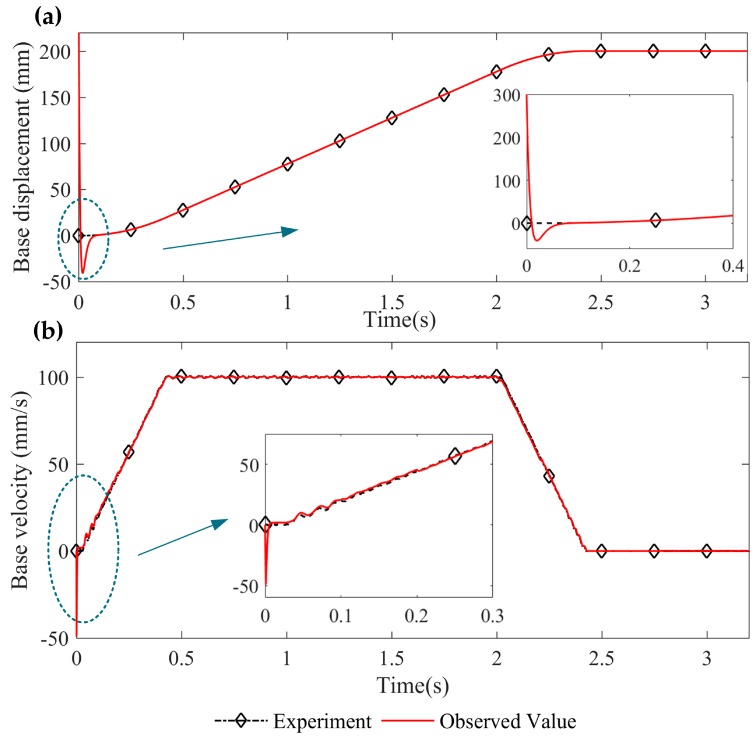
Base displacement and velocity observation: (**a**) base displacement tracking and (**b**) base velocity tracking.

Figure 8 shows the tracking effect of the base displacement and velocity by the designed speed observer. It can be seen that the speed observer can accurately track the base displacement and velocity under the movement of constant acceleration, constant speed, and constant deceleration. The tracking speed of the base displacement is high and the observation deviation of the base velocity can be eliminated rapidly. 

**Figure 9:**
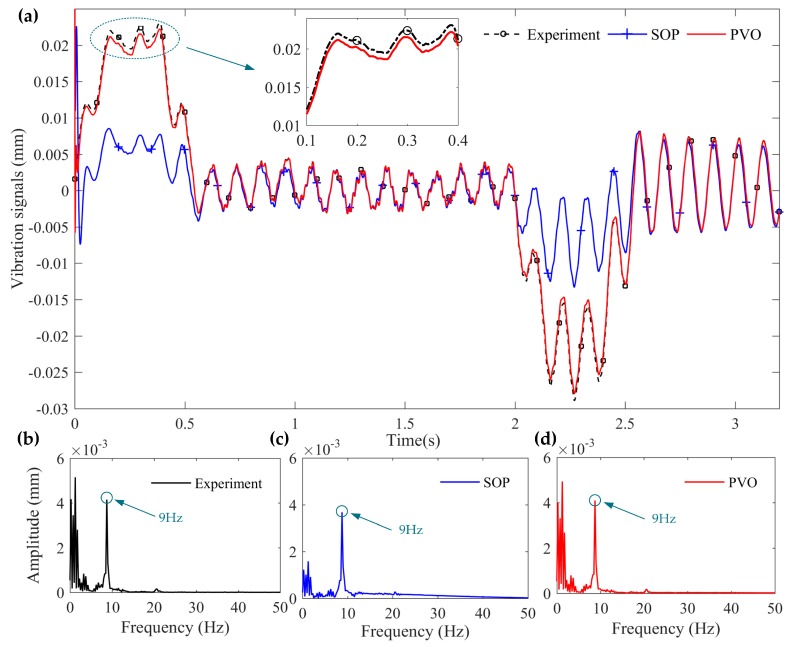
Tracking effect of the vibration signals (*x* = 150 mm): (**a**) tracking effect in time domain; (**b**) amplitude spectra of the experiment results; (**c**) amplitude spectra of the SOP; and (**d**) amplitude spectra of the PVO.

The vibration signals of *x* = 150 mm are selected to be observed to verify the proposed vibration observer. In order to further show the performance of the proposed vibration observer, a comparison experiment is added in which the observer gains are designed by the pole assignment method. The results are illustrated in Figure 9, where SOP means the estimation of the comparison experiment and PVO indicates the estimation of the proposed vibration observer. Although there are certain errors in the amplitude tracking during the change of the base motion state, the tracking trend of the proposed vibration observer is identical to the experimental results, which is much better than the SOP. More important, when the base stops, the proposed vibration observer can accurately observe the residual vibration of the flexible-link manipulator which is significant for vibration control. Figure 9b–d show the amplitude spectra of the experiment and two observation results for the vibration signals (*x* = 150 mm). It is observed that the dominant vibration frequency of the measured results is close to 9 Hz, which is the first-order natural frequency of the flexible-link manipulator. Although the dominant vibration frequency of the SOP is close to 9 Hz, the errors in the amplitude tracking are much larger than the PVO, especially during the change of the base motion state. Above all, the flexible vibration of the TFM can be well observed by the proposed vibration observer.

**Figure 10:**
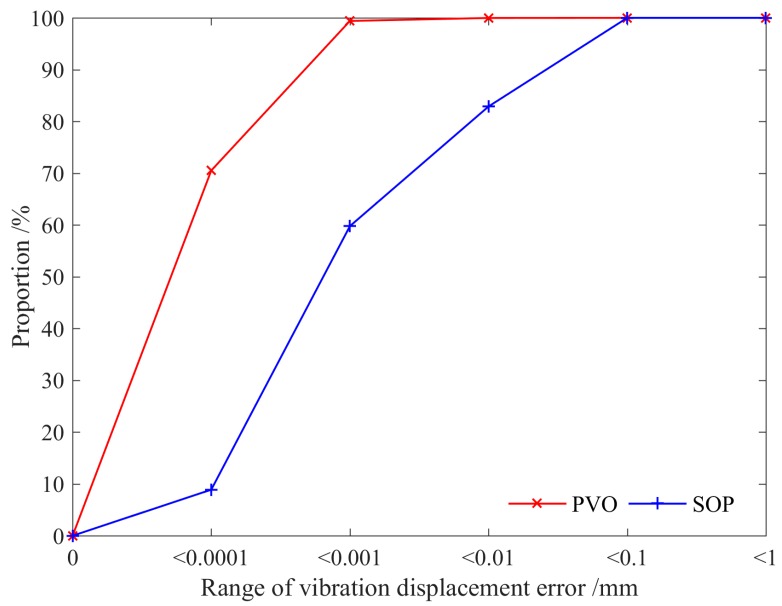
Proportion of vibration displacement error.

Figure 10 shows the statistical investigation and analysis of the vibration displacement errors for the vibration signals (*x* = 150 mm). The PVO method has 70.51% sampling points with less than 0.0001 mm vibration displacement errors and 99.40% sampling points with less than 0.001 mm vibration displacement errors, while the proportions of the SOP method are, respectively, 8.87% and 59.76% at the corresponding vibration displacement errors. Table 1 lists the performance comparison among PVO and SOP techniques. The proposed vibration observer can effectively reduce the mean and variance values of the observation error. It is observed that the effect of the proposed vibration observer is much better.

## 5. Conclusions

In this paper, a two-time scale virtual sensor, based on the decomposition model of the TFM system, is proposed to estimate both position and speed signals of the rigid base and the elastic vibration of the flexible-link manipulator. Through the linear-quadratic differential games and Lyapunov theory, the stability of the two-time scale virtual sensor is verified under the optimization of the observer gains. Results show that the speed observer can exactly estimate the velocity of the base and the vibration observer can accurately observe the elastic vibration signals, as well as their time derivative of the flexible-link manipulator under the motions of constant acceleration, constant speed, and constant deceleration. Through this procedure, the number of the additional position sensors can be effectively reduced and the speed sensors are unnecessary, which has positive significance for enhancing the system reliability and reducing the cost. Moreover, the global motion of the rigid base and the elastic vibration of the flexible manipulator can be estimated by an independent observer, which can effectively solve the rigid-flexible coupling of the TFM and improve the timeliness of the observer. The proposed observer is of great importance for the elastic vibration suppression of the flexible beam structure by PZT actuators.

## Figures and Tables

**Table 1:** Performance comparison among SOP and PVO techniques.

Performance	SOP	PVO
vibration displacement error range/mm	[−0.048, 0.016]	[−0.048, 0.008]
Mean of vibration displacement error/mm	2.89 × 10^−4^	1.14 × 10^−5^
Variance of vibration displacement error	4.05 × 10^−5^	7.62 × 10^−7^

## Supplementary Materials

The following are available online at http://www.mdpi.com/1424-8220/16/11/1804/s1.
